# Observations on Hereditary Hemorrhage

**Published:** 1828-11

**Authors:** Reynell Coates


### HEREDITARY HEMORRHAGE.

*Observations on Hereditary Hemorrhage.* By REYNELL COATES, M.D.

(Concluded from page 315.)

*Remarks.*—Whether the effects of pressure are similar in all cases of this strange idiosyncrasy, is a question that cannot be determined from the limited evidence which is at present within my reach; but it is evident that, among the relations of Mr. W., it is not only unavailing, but even productive of much inflammation, and in some instances of extensive ecchymosis. In the case just narrated, it will be perceived that pressure, in common with many of the styptics employed, exerted some control over the discharge upon its first application; while it eventually produced an exacerbation, by increasing the local irritation.

During the first few days the patient ate but little, and, as he was at the same time freely purged, his strength rapidly declined; but, after a generous course of diet was instituted, the disease became more manageable. It was curious to observe how regularly the hemorrhage intermitted after breakfast, which was his most considerable meal. This effect was probably owing to the concentration of blood about the stomach, during digestion, acting in the manner of a counter irritant. A plentiful supply of food appeared absolutely necessary to support the patient under the immense drain to which he was subjected; but the effect of the tonic and stimulant remedies, resorted to towards the close of the case, sufficiently prove that, however great the debility may be in such cases, no benefit is likely to result from any thing which tends materially to increase the activity of the general circulation.

The actual cautery, so generally successful in former injuries occurring in this family, failed entirely in the present case; and it is worthy of remark, that the temporary benefit resulting from it became less apparent in proportion to the severity with which the process was conducted.

The employment of cauterising irons is so exceedingly unpopular at the present day, that many surgeons have scarcely witnessed it: they are, therefore, generally ignorant of the fact that the application of the iron at a clear white heat is productive of little pain, and almost no inflammation; but that, when the temperature is less considerable, much pain and irritation are immediately produced, followed in many cases by severe inflammation.

An iron, small enough to enter the socket of a tooth, will inevitably lose much of its heat between the fire and the mouth. An additional portion will be dissipated by the moisture of the part before it can act upon the solids; while the steam resulting from the application, together with the radiant heat of the iron, must necessarily stimulate the neighbouring surface, in defiance of every precaution. The evident exacerbation of symptoms produced by the cautery in Mr. W.'s case resulted, no doubt, from all these causes combined, and should caution us against depending too much upon it in such affections.

The phenomenon which presented itself after the final application of the iron, (I allude to the escape of blood from the uninjured gum,) clearly demonstrated that a wound of any kind is not in all instances necessary to the production of this disease. One of the cases mentioned in the editorial notes upon the paper of Dr. Humphrey goes to establish the same fact, and appears to prove that some affinity exists between this complaint and purpura hemorrhagica.

The case is extracted from a communication by Dr. Elssaesser, published in Hufeland's Journal for February 1824. The patient was constantly subject, from the time of dentition, to numerous subcutaneous extravasations of blood, forming maculæ, from the eighth of an inch to an inch in diameter, which disappeared in five or six days, after undergoing the usual changes of colour, giving place to fresh eruptions in other spots. This patient fell a victim, in childhood, to a hemorrhage caused by a slight wound on the head. His two brothers evinced the same strange idiosyncrasy.

It does not appear that any of the cases to which I have alluded have been attended with hemorrhage from considerable arteries: on the contrary, the blood seems to ooze from the capillaries alone. Among the descendants of Mrs. Smith, the discharge generally commenced several days after the injury, at a time when the wound was partially healed. In the sickle-cut which Mr. W. received, I have reason to believe, judging from his own description of the symptoms, that no unusual amount of arterial bleeding occurred; and, in Dr.

Elssaesser's case, death was occasioned by the accidental removal of a scab from a very slight cut, situated on the patient's head.

From these facts, it would appear that this peculiar flux, even when occasioned by a wound, has nothing in common with simple traumatic hemorrhage. It is, therefore, less surprising that the ingenious contrivance suggested by Dr. Physick was not productive of the same benefit in this case which had resulted in those wherein he had previously employed it. The permanent extension of the cheeks, and the action of the air, stimulated the salivary glands, and increased the activity of the circulation in the neighbourhood of the injured part. The coagulation of the blood on its exit, by means of the spunk, was of no avail. Indeed, whenever the wound was left undisturbed for any considerable time, the whole surface would be found covered with a dense coagulum, but this did not lessen in any degree the rapidity of the discharge.

I would not, however, be understood to say, that the prevention of suction was unimportant in the case of Mr. W. On the contrary, I think it highly probable that the disease proved less obstinate in consequence of the slight separation of the incisors, which was kept up by the cork introduced between them. Over this the lips were closed; involuntary suction was prevented, and the salivary glands and fauces were not stimulated to undue action. But the successful termination of this formidable affection is, I think, mainly referrible to the timely adoption of an invigorating plan of treatment, and the free employment of the tincture of opium. Indeed, I am convinced that, had all stimulant and local treatment been omitted on the 27th of December, the case would have terminated more speedily.

Since the preceding pages were written, I have met with several other notices of hereditary hemorrhage, in which are contained facts interesting in themselves, and illustrative of the observations contained in the present communication.

An essay, by Dr. John Hay, of Reading (Mass.) published in the N. E. Journal, vol. ii. p. 221, gives a very full account of this idiosyncrasy, as it occurred in Mr. Oliver Appleton, of Ipswich, and his male descendants, through many generations. One of this gentleman's relations married a Mrs. Smith, of Haverhill, supposed to be the lady alluded to in Dr. Otto's first paper on this subject.

As many of the sufferers were members of the medical profession, there is every reason to suppose that all the resources of the art were properly tested; but it does not appear that any check was opposed to its fatal career until the trial of the sulphate of soda.

Cases are mentioned of bleeding from almost every species of slight injury; but the most important facts are as follows:

The predisposition does not display itself in every generation, but descends through the grandsons in the female line. "The bleeders" are all of a light complexion, apparently healthy, and of a choleric disposition. The hemorrhage seldom appears for several days after the wound, never flows *per saltum,* and is sometimes accompanied by considerable pain. A wound is not in all instances necessary to the discharge; for death has occurred from epistaxis and hæmoptisis; and, in one instance, two quarts of blood flowed from the integuments of the hand, after a slight bruise which did not abrade the skin. When ligatures have been applied above a wound to arrest the discharge, it has burst forth again, from above the ligature, attended with much pain.

Dr. Hay mentions that, during the discharge, the "bleeders" are very much troubled with costiveness and vomiting. He states that the sulphate of soda, given in the dose of an ounce, and repeated daily for several days, generally proves successful; but that, when employed "more frequently," it never fails: he mentions, however, but one case in which it was attended with advantage.

Drs. William and Samuel Buel have published, in the N. Y. Physico-Med. Journal, vol. i., an account of a similar family predisposition in the descendants of the Rev. Timothy Collins, the first pastor of the town of Litchfield, Conn. His son was the first who displayed it, and it afterwards affected some of the branches of the family in every generation, confining its attacks to the males. One of the fatal cases resulted from the extraction of a tooth. The hemorrhage never flowed *per saltum,* and the blood always coagulated freely. Those who were affected were of sallow complexion, and subject to plethora and spontaneous hemorrhage from the nose and mouth. No mechanical or styptic treatment was productive of any benefit. Pressure usually "distended the part above, rendering it livid and intolerably painful; nor could relief be obtained until the discharge was permitted to continue."

Dr. Elssaesser's papers* contain several interesting cases; but it is unnecessary to present them to the reader, after what has been already narrated. Two of the individuals affected were sisters; and these, with one other, are the only

* Hufeland's Journal, February and September 1824.

exceptions that I have met with to the general rule that confines the disease to males. These females were subject to spontaneous hemorrhage in early life.

Dr. E. has also communicated a post-mortem examination of one of his cases. The body was found "bleached" in almost every part. The heart and large vessels discharged a thin and slightly reddish serum. There was a marked deficiency of secretion in the serous cavities. Many glands in the mesentery, and several in front of the lower part of the trachea, were enlarged. The cardiac half of the stomach was "uniformly pale red." The upper portions of the small intestine were pale, but, from the commencement of the ileum to the anus, the canal was reddened; the depth of tint increasing as it descended. This portion contained mucus, slightly coloured in the ileum, but mixed with florid blood in the rectum. The case commenced with hemorrhage from a slight wound, and terminated by discharges of blood from the rectum.

Professor Nasse, of Bonn, has published an elaborate paper on this subject.* The original I have not seen, and the slight notice of it contained in the N. E. Journal furnishes no additional information.

I might refer to many other isolated cases of this disease, and also to a few more instances of family predisposition to it,† but it is time to close this paper, with a few practical remarks.

Hereditary hemorrhage is a disorder so rare, that very few have enjoyed the opportunity of witnessing a sufficient number of cases to warrant them in adopting general views of its pathology; and, unfortunately, the recorded facts are not so numerous as to supply the deficiency. It must be left for others, at some future time, to detail with accuracy the morbid appearances, and peculiarities of temperament, which characterise the "bleeders." Setting aside all speculation, I shall content myself with offering a few comments upon the preceding cases, and some practical conclusions deduced from their history.

All the evidence before us tends to shew that no great aberration from the customary state of health is necessary to produce this hemorrhage in the "bleeders." In many instances, no wound or obvious lesion of any part is necessary to the attack. The blood-vessels appear to be at all times ready to pour out their contents upon the slightest excite-

* Horn's Archives, May and June 1820.

† Vide Ed. Med. and Surg. Journal, vol. iv. p. 513; and N. E. Journal, vol. vi. p. 235.

ment. The family at Ipswich were robust and choleric, characteristics very consistent with an undue degree of plethora and activity of the circulation. The "bleeders" of Litchfield were sallow and less vigorous, but were constantly liable to attacks of plethora and local determinations of blood, which were generally relieved by epistaxis or other hemorrhages.

The state of the circulation in the case of Mr. W. is very peculiar. His cheeks are generally red, and the other parts of his face pale; but his colour constantly varies with the play of his feelings. His pulse ordinarily beats with an active stroke, and there are commonly about eighty-five pulsations in the minute. When he is engaged in earnest conversation, its rapidity is considerably increased; and I have known it to rise from eighty-two to ninety-five, from the exertion of walking across the floor.

In the worst forms of this idiosyncracy, in Dr. Elssaesser's case, for example, irritations, too slight to be discovered, are sufficient to produce extravasation.* Where this disposition is less marked, a local congestion, as in some of the Litchfield cases, or a bruise which does not abrade the skin, as in one of Dr. Hay's cases, may suffice; but more commonly a wound is required to call the disease into action.

It is obvious, however, that few of the "bleeders" would arrive at years of discretion in the midst of so many dangers, if they were at all times equally liable to hemorrhage. It will be remembered that Mr. W. once received a severe wound, which was productive of no serious consequences. It is, therefore, a matter of considerable importance to guard those who are subject to this disease against all causes of plethora; and, among these, suppression or diminution of the natural secretions is the most powerful.

Dr. Hay particularly mentions that his cases were affected with great costiveness and irritability of stomach, during the time of the discharge. Such a condition of the bowels could hardly fail to increase the liability to hemorrhage in persons so constituted, and also to render it more obstinate when it occurs, by closing the most important flood-gate of the circulatory apparatus, if I may be allowed to employ such a simile.

The condition of the hepatic secretion is also of the highest importance upon the same principle.

In the fall of the year 1824, I attended a woman who had laboured under jaundice for two years. She was attacked

* See a case of bloody sweat in a young girl. By MESAPORITUS. Phil. Trans. Abr. vol. v.

by rheumatism, for the relief of which I performed acupuncture in front of the arm, over the middle of the radius. Blood flowed from the orifice formed by the needle, when not restrained by a compress, and continued to do so until the time of her death, which happened three days afterwards.

While the present paper was in preparation, Dr. CONDIE had the politeness to furnish me with some notes of a somewhat similar case, which occurred in his practice in 1827. The patient O'M. had been affected with jaundice for some time, and every part of his skin was of a deep yellow tint, inclining to green. He had been intemperate for some years. Dr. C. was called to see him on the 23d of June, and found him affected with much derangement of the stomach, costiveness, loss of appetite, and other symptoms of hepatic disease; all which, excepting the colour of the skin, subsided considerably by the 5th of August, under the usual treatment. On the 7th, he complained of burning and tickling of the gums, with a slight discharge of blood from them. The hemorrhage increased, and by the next morning became very considerable, although there was neither wound nor alteration of structure in the part. It yielded readily to a combination of opium, ipecacuanha, and acetate of lead. On the 9th, the discharge returned, and flowed from the mouth in a stream. It coagulated beneath the tongue, the coagula forming complete casts of that part of the mouth. When the gums were wiped with a sponge, the blood was seen to start up at every pore from the whole surface. The opium, ipecacuanha, and acetate of lead, were continued, and cold water was held constantly in the mouth; and, when the patient became extremely exhausted, wine and water were administered. By these means the hemorrhage was checked; but, a severe diarrhœa having supervened, death took place on the 10th. The examination of the body was not permitted.

*Deductions.*—Depletion seems to be imperiously demanded in the first onset of this disease, whenever there exist any marks of general plethora; but, with regard to the choice of the means, considerable caution is required.

Venesection is hardly admissible; for cases are not wanting in which the incision made by the lancet has formed a new centre for the hemorrhagic action.*

Purgatives are decidedly proper, even in the last stages of the complaint, where there is any tendency to costiveness;

* See the case already cited in Phil. Trans. vol. v.

for by such means we establish a natural flow of secretions, the suppression of which cannot fail to be injurious.

Calomel may be indicated where there is deficient or morbid action of the liver; but, in employing this article, we should be careful to recollect that ptyalism is very readily induced in patients who have lost a large amount of blood; and such an accident could not fail to induce unpleasant consequences in this disease.

Sulphate of soda, which is said to have had a specific effect in hereditary hemorrhage, certainly deserves especial attention, as the only successful remedial measure heretofore communicated to the public; but I should be more willing to place its effect in the Ipswich cases to the credit of its purging properties, than to any peculiar power that it can possess.

Tonic remedies are indispensable at the close of the disease, when extreme exhaustion is induced by the discharge; but those only which appertain to the class of dietetics are unexceptionable.

Direct stimulants are altogether inadmissible.

Narcotics are strongly indicated, as exercising a powerful influence over irritation; and in those cases occasioned by a wound they are all important, in consequence of the inefficacy of local treatment: they should be administered liberally.

Local measures are generally unavailing, with only one exception.

Astringents are invariably followed by an ultimate increase of the flux, though they may lessen it for a short period.

Pressure is productive of irritation and inflammation without suppressing the discharge, which, when denied an outlet, will produce either an internal extravasation, or a new hemorrhage from some other part of the body.

Cold was thought to produce some benefit in Dr. Condie's case; but it was not applied alone, and the remedy was fully tested in the case of Mr. W., but without advantage.*

The actual cautery has succeeded in many of the lighter cases; but it should never be persevered in after it has been once properly applied without advantage.

Potential cautery has been employed in one mild case only, and in this case it was unsuccessful. From what I have seen of its effects in traumatic hemorrhage from small vessels, I should be inclined to view it favorably.

These deductions are not offered to the public as established facts. The ground is in great degree new, and the amount

*** Ice was applied to the gums during one night, but unfortunately no note of the fact was preserved.**

of experience small. My remarks are thrown out in the hope that some advantage may result from placing what is known more immediately before the profession, and from the proofs here given of the inefficacy of the usual plans of treatment. They must be subjected to the test of future observation.

